# Depression and Sequential Decision-Making Revisited

**DOI:** 10.3389/fpsyg.2019.01492

**Published:** 2019-07-01

**Authors:** Martha Sander, Steffen Nestler, Boris Egloff

**Affiliations:** ^1^Department of Psychology, Johannes Gutenberg University Mainz, Mainz, Germany; ^2^Department of Psychology, University of Münster, Münster, Germany

**Keywords:** decision-making, major depressive disorder, secretary problem, sequential decision, punishment sensitivity

## Abstract

**Background:** The effect of depression on decision-making is an important but still an unsettled issue. Although most studies have reported that clinically depressed participants show worse performance, there are also studies that have shown no or even positive effects. Specifically, von Helversen et al. (2011) were able to document a positive effect of depression on task performance in a sequential decision-making task called the secretary problem (SP). Here, we (1) aimed to replicate this study in an extended version using more trials and (2) modified it by including an additional condition in which negative feedback was given.

**Method:** Eighty-two participants took part. They were split into two groups: 20/21 participants with major depression disorder (MDD) and 20/21 matched healthy participants. Participants completed the secretary problem either in the standard or in a modified version. Additionally, they answered questionnaires for assessing depression, personality, and intelligence.

**Results:** We did not find any significant differences between clinically depressed and nondepressed individuals in any indicators of task performance, under both the original and modified conditions.

**Limitations:** Our participants were ambulatory patients. The quality of depression may have been therefore less extreme. We did not assess or control for rumination.

**Conclusions:** We were not able to detect any significant differences between the performances of healthy and clinically depressed participants in a sequential decision-making task.

Imagine you are going to speed-date events. You have the aim to find a partner who fits the best to you. You get to know one person after another for a few minutes, and before a bell rings, you have to decide if you want to get to know the actual potential partner better or to move forward to the next one. Importantly, you do not know if the subsequent potential partners fit better or worse. Once rejected, it is unlikely you will get to know that specific person later on as she/he might have found someone else or might have left disappointed.

This scenario illustrates a sequential decision-making task. Sequential decisions are usually categorized into the broader class of dynamic decision-making (DDM; Steyvers et al., 2008). DDM is characterized by a series of interdependent decisions, the fact that the state of the task may change, autonomously or due to the decisions of the decision maker, and that decisions have to be made in real time (Brehmer, 1989)^1^. The reason why sequential decision-making is usually classified as DDM (see Gonzalez et al., 2017) is that the decisions that are made influence future decisions according to their outcome and that the state of the tasks varies (autonomously). DDM usually refers to rather complex tasks that can be represented in computer simulations where they are called microworlds (Brehmer and Doerner, 1993). A typical task, for example, is commanding a group of firefighters in real time to fight a forest fire which is about to spread out, influenced by the weather and the consequences following the decisions made by the decision maker. Compared to that, sequential decision-making is seen as a simplified DDM task that regularly involves less variables. According to Gonzalez et al. (2017), individuals show the same complexity and dynamics in behavior, whether they work on classic complex DDM tasks or on simpler DDM tasks that only involve certain dynamic complexities like sequential decision-making tasks. In contrast to DDM, static decisions are defined by the fact that only one decision has to be made overall (see Wang and Ruhe, 2007). Furthermore, decision tasks can be divided according to the degree of certainty they provide, ranging from uncertain (not even the outcome of a decision is known), ambiguous (the outcomes are known but not the probability of them) to risky (the probabilities of outcomes are defined) and certain (only one outcome is possible and known; Starcke and Brand, 2012).

Most decision-making tasks used in research, being static or dynamic, involve risky or ambiguous problems. Investigating the performance of individuals in these tasks led regularly to results showing that participants did not perform in accordance with a theoretically optimal strategy. In case of the firefighting microworld, for example, participants very often let the headquarters of their fire station be burned down, what should have been avoided (e.g., Brehmer and Allard, 1991). To explain the performance of individuals in these tasks, many theories have been proposed. Doerner (1989) assumes decisions are made as fast as possible (like “cannonballs”) without caring too much for the (often not optimal) result. Others argue that decisions are not usually made in a rational or strategic but rather in an *intuitive* way by relying on heuristics. According to Tversky and Kahneman (1974), heuristics enable fast decisions that may, however, be susceptible for mistakes when compared to a normative standard (e.g., probability theory). On the other hand, Gigerenzer and Todd (1999) argue that heuristics enable decisions with minimal information that are as good as more complex decision-making strategies. Independent of their success, heuristics are often tied to affective processes. Damasio (1996)’s somatic marker hypothesis, for example, emphasizes the role of emotions in the decision process by assuming that decisions activate somatic states that are associated with rewarding or punishing experiences that had been made in the personal history after similar decisions (Starcke and Brand, 2012). These somatic markers may influence the actual decision-making behavior by biasing the weighing of the available information used during the decision-making process (Buelow and Suhr, 2009) and enable individuals to decide in a fast way without requiring an elaborate and intensive cognitive analysis of the available decision options. Especially in complex decision-making tasks, these somatic markers are assumed to help deciding faster and acting adequate. Thus, the experience of and sensitivity to rewarding or punishing feedback should have a significant influence on further decisions.

Furthermore, we believe that these processes should be more prevalent in sequential decision-making tasks, as feedback about the quality of the actual decision is presented immediately, contrary to other rather complex DDM tasks which often contain delayed feedback (see above, but also Brehmer, 1989). In addition, the impact of punishing or rewarding feedback on decision-making can be easily examined and also varied in different ways in sequential decision-making tasks (compared to classic DDM tasks; see Brehmer, 1989).

In sum, sensitivity for reward and punishment may play an important role in explaining the performance in sequential decision-making tasks (beside other factors like the sort of used heuristics, complexity of the task, and personality traits that refer to risk behavior, see Steyvers et al., 2008). Hence, it may be useful to investigate the variables that are related to the sensitivity for reward or punishment to better understand performance in sequential decision-making task. We believe that having a major depression disorder (MDD) is such a variable (e.g. Cella et al., 2010) that influences sensitivity for reward and punishment and therefore investigated the decision-making behavior of individuals with MDD in a sequential decision-making task.

With an estimated lifetime prevalence of 14.6% (Bromet et al., 2011), MDD is one of the most common psychiatric diagnoses worldwide. The main symptoms of MDD are a nearly permanently depressed or irritable mood, identified either through a subjective account or by others, and a decrease in interest or pleasure in most of the person’s usual activities nearly every day for a period of 2 weeks (American Psychiatric Association, 2013). Also, patients often report a lack of concentration and diminished decision-making abilities. Because of the frequent occurrence of the last symptom, it was added as a relevant criterion for depression in the DSM-5 (“Diminished ability to think or concentrate, or indecisiveness”; American Psychiatric Association, 2013). Although the DSM-5 does not explicitly state whether indecisiveness refers to a reduction in the number of decisions or a worse quality of decisions, most research has explored differences between healthy and persons with MDD in the quality of their decisions (see, e.g., Must et al., 2006; Cella et al., 2010).

In most studies to date, the Iowa Gambling Task, a type of DDM, (IGT; Bechara et al., 1994) was employed as a sequential decision-making task to examine differences in the quality of decision-making. The IGT was originally created to assess real-world decision-making for individuals with orbitofrontal cortex damage (Buelow and Suhr, 2009). The original version of the IGT consists of five blocks of 20 trials. In each trial, participants have to decide which card to select out of four concurrently available decks for monetary gain or loss. The first two decks are associated with a high constant gain but also with a high potential loss that occurs after an unknown number of selections from these two decks by the decision maker. Selecting these decks lead, in sum, to a cumulative long-term loss. The remaining two decks are associated with less immediate reward but also with fewer losses, and thus, selecting them results in a cumulative long-term gain. Each time, the participants choose a card, they get verbal feedback (on screen) about winning and/or losing some money. Before the start of the task, participants are informed that every card is associated with gaining a certain amount of money, but for each card chosen, there is also a 50% chance of losing some money as well. Given these arrangements, it is thus wiser to select the latter two decks. It is interesting, however, that Cella et al. (2010) found that a sample with MDD performed worse than a healthy sample due to their more frequent selection of the two disadvantageous decks. In addition, when the originally advantageous decks were gradually replaced with the disadvantageous decks, the clinically depressed participants responded less quickly to this change than the healthy controls did. Other studies have confirmed these results, indicating worse performance in different cognitive and decision-making tasks for persons with MDD compared with healthy persons (Austin et al., 2001; Steffens et al., 2001; Must et al., 2006; Adida et al., 2011; Blanco et al., 2013).

One explanation for these findings is the assumption that individuals with MDD are characterized by an altered sensitivity to reward and punishment (c.f. Cella et al., 2010). Some researchers have suggested that clinically depressed individuals are hyposensitive to reward (Henriques and Davidson, 2000; Bylsma et al., 2008; Eshel and Roiser, 2010), which in turn may lead to lower levels in associated pleasant feelings (Martin-Soelch, 2009). According to Cella et al. (2010), participants with MDD might fail to change their behavior because they are not sensitive to changes in reward contingencies and thus achieve a poor adjustment to a changing environment.

It is interesting, however, that in other studies, persons with MDD have performed as well as healthy individuals. Dalgleish et al. (2004) (see also Kyte et al., 2005), for example, found that there were no significant performance differences in the IGT between a clinically depressed sample and a healthy control sample. In other studies, individuals with MDD even outperformed healthy persons (Alloy and Abramson, 1979; Forgas, 1998; Au et al., 2003; Storbeck and Clore, 2005; Smoski et al., 2008). In Smoski et al. (2008), for instance, individuals with MDD achieved better results on the IGT than healthy controls. They took fewer cards from the disadvantageous decks, and from the beginning, they made better choices. To understand the procedure of this and similar studies using the IGT as a paradigm, we want to give some insight. In the study of Smoski et al. (2008), participants were read the instructions by the experimenter. They were briefed to choose cards one at a time from any of the four available decks. They were able to switch form one deck to another at any time and as often as they wanted. All participants were informed that all cards would lead to a monetary win but that some cards would in the same time lose them money. In sum, they might lose more money than they won in the actual trial. The participants were also told that some decks are worse than others and that it is possible to win in the end if they stayed away from the worst decks.

The authors claimed that depression is associated with higher responsivity to negative feedback (which means in this case losing money in a trial; i.e., punishment) relative to reward (in this case if the trial led only to a monetary gain) and that persons with MDD are hypersensitive to negative feedback and punishing stimuli (Henriques and Davidson, 2000; Bylsma et al., 2008; Eshel and Roiser, 2010).

The increased sensitivity to negative feedback and punishing stimuli (Elliott et al., 1996) seems to lead regularly to a lower performance, manifested in more errors committed in trials in which negative feedback was given (Beats et al., 1996; Steffens et al., 2001; Nestler and Carlezon, 2006). Nonetheless, Smoski et al. (2008) claimed that individuals with MDD are more risk-averse and may have an increased sensitivity to aversive contingencies than control participants without MDD. If true, this would lead to a faster learning of contingencies to avoid failures. In the IGT, individuals with MDD would thus adopt a low-risk low-reward strategy (Smoski et al., 2008; Paulus and Yu, 2012) and would show enhanced attention to negative feedback.

Even though the IGT is a very interesting and rather simple way to examine the decision-making of individuals, it also has some shortcomings, especially with regards to the processes the task is hypothesized to measure (Buelow and Suhr, 2009). At first, it was thought that the IGT measures “hot” decision-making, which involves emotional and affective responses to the options (Bechara et al., 1996). However, later empirical research showed that participants seem to learn the win/loss contingencies of the IGT so that the first IGT trials measure decision processes under ambiguity, while the later trials assess processes underlying risky decisions (Brand et al., 2007); both of them involve rather “cold” decision-making which is associated with rational and cognitive determinations of risks and benefits of options and the ability of holding them in mind and contrasting them. Furthermore, it is still unclear whether and how mood, personality traits, or intelligence affect participants’ performance in the IGT (Buelow and Suhr, 2009). Altogether, this might explain why the results vary that much between the different studies on the performance of clinically depressed patients in the IGT.

Using a different sequential decision-making task, the so-called secretary problem (SP), von Helversen et al. (2011) showed that clinically depressed individuals performed better in a sequential decision-making task compared with healthy individuals. In contrast to the IGT, participants do not have to choose between four potential opportunities at the same time in the SP. Rather, they have to decide sequentially if they take an option in that moment or not. Even though the two tasks differ in their experimental set-up, they both are categorized as DDM tasks (see Steyvers et al., 2008). Also, both tasks examine how human decision-making is influenced by feedback. The goal of the present study was to replicate and extend von Helversen et al.’s (2011) findings. Therefore, we start with briefly describing the SP and the advantages of using this task.

## The Secretary Problem

The SP (Ferguson, 1989) was designed to investigate the performance of people in the case of sequential decision problems like the one at the beginning of the article. Regularly, in the SP, the decision maker’s duty is to select the best alternative from a series of presented alternatives. In a famous and classic variant of the SP that is often used in research, participants are asked to put themselves in the role of a company manager who needs to hire one secretary. They know that *N* persons have applied for the position; that there is a best, second best, third best, and so forth applicant; and that their task is to hire the best applicant. To this end, applicants are presented sequentially, and for each applicant, the decision maker is informed about the applicant’s relative rank, that is, whether she is the best, second best, third best, and so forth for the job compared with the *previously* seen applicants. After each applicant, participants have to decide whether they will hire this applicant or not. Furthermore, once rejected, an applicant cannot be recalled. In this version of the SP, participants receive a (monetary) reward only after selecting the best applicant.

Using the SP has a number of advantages: First, it is a relatively easy decision-making task because only the relative rank of the applicants has to be considered. Due to this simplicity, it also allows to investigate basic decision-making processes (see Gonzalez et al., 2017). Second, the SP has an optimal solution that can be mathematically determined (see Ferguson, 1989): According to this *threshold* strategy, decision makers should examine a certain number of applicants first until a threshold of examined applicants, *r*, is reached. Thereafter, they should select the next applicant who has a relative rank of 1. Thus, the first *r* applicants should always be rejected, and the decision-maker should then hire the first applicant out of the pool of *N* − *r* available applicants with the relative rank of 1. The quotient *N/e* (*e* names Euler’s number) can be used to estimate the threshold *r* for an arbitrary number of *N* participants. For example, if the number of applicants is *N* = 40, the optimal strategy states that one should reject the first 16 applicants and then, out of the remaining 24 (*N* − *r*) applicants, one should select the first one with a relative rank of 1. In the case that also the second and third best candidates will be rewarded (rank-dependent pay-off structure; see Bearden et al., 2006), a multiple threshold strategy is mathematically the best strategy to be applied. This strategy states multiple cut-offs that determine when participants should accept an applicant with a relative rank of 1, a relative rank of 2, and so on. In the case of *N* = 40 applicants, for example, the optimal *multiple* threshold strategy assumes that a participant should reject the first 12 applicants (i.e., the first threshold is 12). Thereafter, participants should accept the next applicant with a relative rank of 1. After the 20th applicant (i.e., the second threshold), the decision maker should accept the next applicant with a relative rank of either 1 or 2. Furthermore, after the 26th applicant, the decision make should accept the first applicant who has a relative rank of 1, 2, or 3. We note that the threshold strategy is optimal mathematically because it maximizes the probability that one selects the *absolute* best candidate. For example, when one employs the rule in case of *N* = 40 applicants, the probability of success by using this strategy is about 37%.

Third, given that an optimal performance strategy is available for the SP, researchers have a theoretically justified criterion that they can use to judge the optimality of a participant’s decision behavior. Fourth, a number of studies exist that investigated the decision strategies of naïve participants in the SP (e.g., Seale and Rapoport, 1997, 2000; Bearden et al., 2006). Seale and Rapoport (1997), for example, hypothesized that persons may use (1) the threshold strategy, (2) a candidate counting strategy (i.e., a decision maker only takes applicants with a relative rank of 1 into consideration and chooses the *x*th applicant with a relative rank of 1), or (3) a successive noncandidate strategy (i.e., the decision maker counts a certain number *y* of applicants with a relative rank differing from 1 appearing after the last applicant with a relative rank of 1 and then chooses the next applicant with a relative rank of 1). Using experiments in which empirical predictions based on the decision strategies were pitted against each other, the experimental results showed that participants’ decision-making behaviors could be described best by the threshold strategy; it accounted for more decisions than the two other rules. However, and in contrast to the optimal strategy, individuals tended to stop too *early* while searching for (one of) the best candidate(s) (e.g., Seale and Rapoport, 2000; Bearden et al., 2006). Their performance was thus worse than the optimal strategy as they typically did not wait long enough for the optimal number of applicants.

Altogether, the SP is a comparatively easy and well-defined decision-making task with an optimal performance strategy. Because of this, the decision-making behavior of the participants can be measured and categorized in a rather simple way. Furthermore, the SP can be categorized as a DDM since it meets its minimal requirements of involving a series of choices taken over time to achieve some overall goal (i.e., maximizing the own reward by choosing the best applicants, see Gonzalez et al., 2017). Furthermore, the SP is comparable to many real-world problems that involve finding the balance between exploring uncertain alternatives (to view the applicants in the SP paradigm) and exploiting familiar ones (taking the best applicant after a determined number of applicants in the SP paradigm). According to Gigerenzer and Todd (1999), this is a basic requirement for successful decision-making. Moreover, the performance in the SP should be correlated with cognitive ability (in contrast to the IGT, see Buelow and Suhr, 2009) because – among other things – it places demands on memory by presenting information sequentially [Lee et al., 2004; in contrast, for example, to the Traveling Salesperson Problems (TSP)^2^, see Applegate et al., 2007], and finally, performance in the SP has been found to differ between clinically depressed patients and healthy participants.

### Depression and Performance in the Secretary Problem

In the following, we want to give some detailed insight in the study of von Helversen et al. (2011), who were able to find a better performance of participants with MDD than healthy participants. von Helversen et al. (2011) used the SP to examine the decision-making performance of a DSM-IV diagnosed sample of patients with MDD (*N* = 15), patients who were recovered from MDD (*N* = 12) and a healthy control group sample (*N* = 27; all groups were matched on sex, age, and education). All participants were asked to play 30 SPs, and performance was measured by awarding participants points that depended on the absolute rank of the chosen applicant. Following the procedure of the standard SP, participants were presented with 40 applicants in a single SP. Applicants were presented one after another in a random order and after each applicant, participants were asked to decide if they accept the actual applicant or not. In the former case, the respective SP ended; in the latter case, the next applicant was presented. In line with the standard SP, participants were only informed of the relative rank of the applicant. Participants in von Helversen et al. (2011) received point in accordance with the *absolute* rank of the chosen candidate. These points could be seen in the corner of the screen and were later converted into monetary compensation. The results showed that the sample with MDD performed better than the healthy control group but did not differ in personal goals to be satisfied with, in search length or in the average relative rank of the chosen candidates. Subsequent analyses showed that clinically depressed participants tended to see more applicants compared with healthy or recovered participants, indicating that they utilized higher thresholds. Formal modeling corroborated this finding: participants with an acute MDD waited significantly longer until they decided to accept an applicant.

## The Present Research

Our goal was to replicate and extend von Helversen et al.’s (2011) study by asking individuals with MDD and healthy control participants to participate in the SP. Specifically, one group of individuals with MDD and one group of healthy controls were asked to participate in a version of the SP that was similar to the SP employed by von Helversen et al. (2011). One other group of individuals with MDD and one other group of healthy controls were asked to work on a modified version of the SP in which they received feedback that depended on the candidate they chose. Depending on the absolute rank of the chosen candidate, the text “Unfortunately you have chosen the *x*th candidate overall. *xx* points out of 40 obtainable points will be subtracted from your score” was presented for 7 s when the chosen candidate was not the absolute best applicant. When they had chosen the best candidate, they read “You’ve chosen the best candidate. No points will be subtracted from the maximum of 40 points.” This modification was introduced to test whether a hyposensitivity to reward or a hypersensitivity to punishment (Cella et al., 2010) is responsible for the better performance of individuals with MDD in the SP. The reasoning behind this manipulation was that the altered sensitivities should make participants with MDD react in a hypersensitive way to the punishment. Therefore, they should learn more quickly to avoid failure by using a threshold strategy that matches better to the optimal strategy in their decision-making. Specifically, we thought that they wait longer to avoid the negative feedback, resulting in higher cut-off values as suggested by von Helversen et al.’s (2011) results. In contrast, performance of healthy participants in the SP should not vary across conditions (i.e., should be independent of feedback, see Campbell and Lee, 2006).

Additionally, we also examined if we could confirm the positive association among intelligence and performance in the SP (Burns et al., 2006) and, for exploratory reasons, if the Big Five personality traits would be correlated in either way with performance in the SP (see e.g., Lauriola and Levin, 2001; Brand and Altstötter-Gleich, 2008).

## Materials and Methods

### Participants

Eighty-six participants took part in the experiment. Four participants (one control and three clinically depressed patients) were excluded from the sample because they misunderstood the sequential decision task.

The final sample of participants with MDD consisted of 41 actual patients who were treated in a university outpatient clinic in Germany. Their mean age was *M*_age_ = 36.5 years (SD = 11.2) and 35 of them were women. Most of the participants finished school with an A level (German “Abitur”; 53.6%). All other participants visited secondary school (“Realschule” in the German vocational system). General inclusion criteria for participants with MDD included a diagnosis of depression according to DSM-IV that was assessed by each patient’s individual therapist on the basis of a SCID-I Interview (Wittchen et al., 1997; American Psychiatric Association, 2007). The first author of this study checked the diagnosis with a screening checklist from the SCID-I Interview and the IDC-L Checklists (Hiller et al., 1995). Furthermore, participants were included when they had an age of 18–65 years, a sufficient command of the German language, and a signed informed consent form. General exclusion criteria consisted of the existence of (1) a current addiction, (2) all forms of schizophrenic disorders, (3) the adult form of ADHD (attention-deficit/hyperactivity disorder), (4) personality disorders, and/or (5) acute suicidality. Three included participants suffered from a dysthymic disorder, but no included participant suffered from a somatic disorder. All participants with MDD were examined during the exploratory phase of the therapy (i.e., no therapy had taken place). Finally, 20 clinically depressed participants received no antidepressant medication, whereas 21 received antidepressant medication: 10 of them received SSRIs (24%, 1 received an additional tetracyclic antidepressant), 3 received SNRIs (7%; 1 of them received an additional antipsychotic medication), 2 received tricyclic antidepressants (5%), 1 received a tetracyclic antidepressant (3%), 2 received Johanniskraut (5%), and 3 did not specify their antidepressant medication (7%).

The final control group comprised 41 participants (*M*_age_ = 36.0 years; SD = 10.3; 35 females). They were selected form the local community. Participants were included if no mental disorder could be determined in the last 5 years according to the DSM-IV (assessed by means of the screening checklist of the SCID-I Interview) and if they were psychotropic-medication-free. These participants were matched on gender, age, and education to the respective patient group with MDD. Again, most of the participants finished with an A level (“Abitur”; 63.4%); the other participants visited secondary school (“Realschule”).

Participants in the control group and participants with MDD were randomly assigned to two subsamples. With regard to the clinically depressed participants, the first subsample (standard condition) consisted of 20 participants with MDD (*M*_age_ = 37.7 years; SD = 12.2; 18 female), while the second subsample comprised 21 participants with MDD (*M*_age_ = 35.4 years; SD = 10.4; 17 female). The first subsample of the control participants (for the standard condition, CG1) consisted of *N* = 20 healthy participants (*M*_age_ = 37.0 years; SD = 11.1, 18 female), while the second control subsample consisted of *N* = 21 healthy participants (*M*_age_ = 35.1 years; SD = 9.7; 17 female).

### Design and Statistical Approach

The design of the experiment is a 2 × 2 between-subjects design defined by the factor health status (control vs. depressed) and the factor condition (standard vs. punishment sensitive). Accordingly, a 2 × 2 between-subjects analysis of variance (ANOVA) and independent *t*-tests were used to investigate the statistical hypotheses. Furthermore, and to follow the statistical analysis of von Helversen et al. (2011) as closely as possible, we also report the results of respective nonparametric tests. The data and the scripts to conduct all analyses can be retrieved from the Open Science Framework^3^.

### Sample Size Calculation

The effect size in von Helversen et al.’s (2011) study was *d* = 0.55. To replicate this effect with a power of 0.80 and type I error rate of *α* = 0.05, 106 participants would be needed (i.e., 53 participants in the experimental and 53 in the control condition). As we assessed 42 healthy control participants and 44 depressive participants here, the power to detect the effect found by von Helversen et al. (2011) was 0.71.

## Materials

### Sequential Decision-Making Task–Secretary Problem

**Figure 1:**
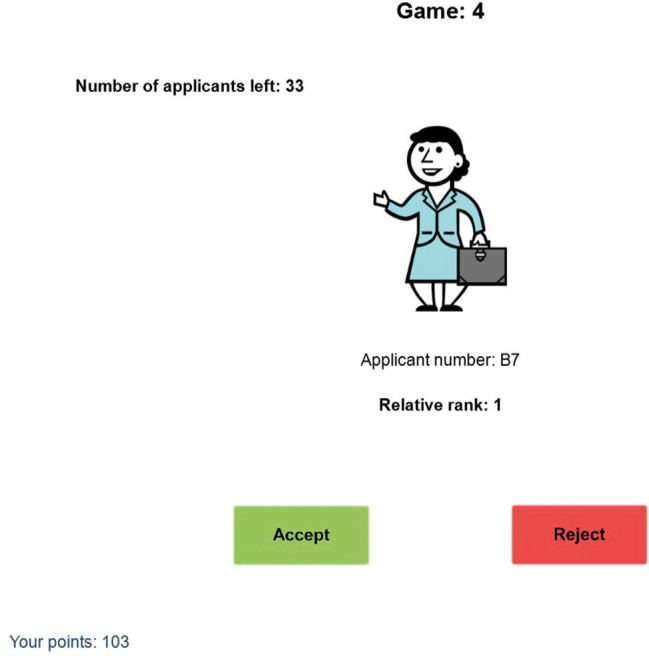
A screenshot of the task. The information participants could use for their decision consisted of the applicant’s number and relative rank.

The SP was conducted as a computer-based experiment. In contrast to von Helversen et al. (2011), participants were asked to play 60 (rather than 30) SP games. For each game, the participant was asked to select the best candidate for a job out of a sequence of 40 applicants. The presentation of the applicants occurred one after another in a random sequence. For each applicant, participants were informed about the relative rank of this applicant. They learned how good the actual applicant was in comparison with the applicants seen so far in the game (see Figure 1). After the presentation of each applicant (see Figure 1 again), the participants had to decide whether they wanted to accept or reject the applicant. If the participant accepted the applicant, the game ended and the next one began. If the applicant was rejected, the next one was presented. This was done until the last applicant was presented; this final one had to be chosen to end the game. Participants were informed that once rejected, that applicant could not be chosen later in that game.

Before each game began, participants were asked to indicate how good the chosen applicant in the game should be to leave them satisfied with their choice. After each game, participants were paid in accordance with the absolute rank of the candidate they chose (see von Helversen et al., 2011). They received 40 points if the chosen applicant was in fact the best one, 39 points for the second best, and so on. At the end of the study, points were exchanged for Euros (100 points = 30 Cents^4^). Note that even when participants received 0 points across all games, they were paid at least 11 Euros. In the punishment sensitive condition, the SP was similar to the SP just described with the only difference that participants received feedback after they had chosen a specific applicant. The text “Unfortunately you have chosen the *x*th candidate overall. *xx* points out of 40 obtainable points will be subtracted from your score” was presented for 7 s when the chosen candidate was not the absolute best applicant. Alternatively, they read, “You’ve chosen the best candidate. No points will be subtracted from the maximum of 40 points” if they had chosen the absolute best candidate.

Following the procedure in von Helversen et al. (2011), we examined the average number of points earned per game (i.e., performance), the average number of applicants evaluated per game (i.e., search length), the average relative rank of the chosen applicants, and the average self-reported goal.

### Questionnaires

Participants were also asked to fill out Beck’s Depression Inventory (BDI-II; Hautzinger et al., 2006), the Big Five Inventory SOEP (BFI-S; Gerlitz and Schupp, 2005), the Multiple Choice Vocabulary Test (MWT-B; Lehrl, 1977) and a short version of Raven’s Progressive Matrices Test (SPM_mod_; Raven and De Lemos, 1958).

The BDI-II is a questionnaire with 21 items measuring symptoms of a depressive disorder. Persons with sum scores ranging from 14 to 19 are considered mildly depressed. Persons with scores ranging from 20 to 28 are considered moderately depressed, and persons with scores between 29 and 63 are considered severely depressed. The reliability of the BDI-II was Cronbach’s *α* = 0.96.

The BFI-S was used to assess participants’ values in neuroticism (N), extraversion (E), openness to experience (O), agreeableness (A), and conscientiousness (C). The BFI-S contains 15 items with three items measuring one of the five Big Five dimensions. For each dimension, we calculated the mean of the items. Cronbach’s *α* reliabilities^5^ of the single scales were E: *α* = 0.85, N: *α* = 0.73, O: *α* = 0.55, A: *α* = 0.60, and C: *α* = 0.80.

Intelligence was measured with the MWT-B and SPM_mod_. The MWT-B is designed to examine crystallized intelligence^6^ of a person using 37 potentially known words that should be differentiated from nonexisting words. The SPM_mod_ is a brief version of the SPM containing 15 matrices from the original SPM (see Raven and De Lemos, 1958). The SPM_mod_ was assessed as a measure of participants’ fluid intelligence.

### Procedure

Our study protocol was approved by the ethical review committee of the Psychological Institute of the Johannes Gutenberg University of Mainz. All participants were examined separately. After signing the consent form, that was in accordance with the Declaration of Helsinki, and after being screened for mental disorders (see above), the participants filled out paper-pencil versions of the questionnaires. They first answered demographic questions including gender, age, education level, and the prescribed medication. Thereafter, they completed the BDI-II and BFI-S. After that, they filled out a computer-based version of the MWT-B and the SPM_mod_. Finally, they worked on the sequential decision task. In the SP, participants were first asked to read the instructions that were provided on the screen and also in a hardcopy. The instructions were designed in accordance with the instructions for the SP described in Seale and Rapoport (2000). After completing two test trials, participants worked on either the standard version of the SP or the version with the punishment sensitive manipulation.

## Results

### Questionnaires

Means and standard deviations for the questionnaires can be found in Table 1. We used a 2 × 2 between-subjects analysis of variance (ANOVA) with the factors health status and condition to examine potential differences between the experimental and control groups.

**Table 1:** Questionnaire measures.

		Standard SP				Punishment sensitive SP			
		Depressed (*N* = 20)		Healthy (*N* = 20)		Depressed (*N* = 21)		Healthy (*N* = 21)	
	Measure	*M*	SD	*M*	SD	*M*	SD	*M*	SD
BDI-II		29.25	8.39	5.95	8.39	27.15	12.98	3.81	2.99
MWTB		30.70	2.92	31.45	4.24	29.71	2.92	29.90	2.62
SPM_mod_		8.20	3.04	7.90	2.86	7.48	3.80	7.33	3.81
BFI-S	Extraversion	3.58	1.63	4.95	1.26	3.81	1.57	4.95	1.16
	Neuroticism	5.73	0.78	4.13	0.91	5.38	1.41	4.27	1.36
	Openness	4.35	1.11	4.77	1.05	4.41	1.28	5.00	1.05
	Agreeableness	5.18	0.95	5.65	0.87	5.25	1.17	5.43	0.92
	Conscientiousness	5.20	1.07	5.68	0.81	4.89	1.64	5.62	0.96

BDI-II, Beck Depression Inventory – II; MWT-B, German version of the Multiple Choice Vocabulary Test; SPM_mod_, short version of Raven’s Progressive Matrices Test; BFI-S, Big Five Inventory-SOEP.

Concerning the BDI-II, we found a significant main effect of the health status factor, *F*(1, 78) = 165.81, *p* < 0.001, *η*^2^ = 0.68, with higher values in the sample with MDD compared with the healthy controls. No main effect of condition and no significant Health Status × Condition interaction emerged, *p* = 0.25 and *p* = 0.99. As BDI-II scores and MWTB scores were not normally distributed, we checked all results using nonparametric versions of the analysis of variance (i.e., Kruskal-Wallis). These analyses yielded essentially the same results as the parametric models.

For the Big Five personality dimensions, we found significant main effects of the health status factor for neuroticism, *F*(1, 78) = 28.24, *p* < 0.001, *η*^2^ = 0.27, extraversion, *F*(1, 78) = 16.01, *p* < 0.001, *η*^2^ = 0.17, openness to experience, *F*(1, 78) = 4.06, *p* < 0.05, *η*^2^ = 0.05, and conscientiousness, *F*(1, 78) = 5.51, *p* < 0.05, *η*^2^ = 0.07. Participants with MDD had higher values on neuroticism compared with the healthy controls (see Table 1 again), and the controls were more extraverted, more open to experience, and more conscientious than the patients with MDD. Neither a main effect of condition nor a Health Status × Condition interaction emerged for these four traits, *p*’s > 0.47. For agreeableness, there were no main effects and no interaction, *p*’s > 0.14.

Finally, we found no significant differences between the participants with MDD and the healthy controls in crystallized intelligence, *F*(1, 78) = 0.44, *p =* 0.51, *η*^2^ = 0.01, or fluid intelligence, *F*(1, 78) = 0.01, *p* = 0.77, *η*^2^ = 0.00. For both variables, neither the main effect of condition nor the Health Status × Condition interaction was significant either, *p*’s > 0.08.

In sum, participants with MDD were less extraverted, less open to experience, and less conscientious while showing higher values on neuroticism than healthy participants. The two intelligence scores did not differ significantly between the two samples.

### Secretary Problem

We now turn to the question whether the two samples differ in their performance with regard to the SP^7^ (see Table 2). We first present the results of the parametric and nonparametric analysis with regard to the means of the performance measures. Thereafter, we report the results with regard to the median of the measures.

**Table 2:** Means, medians, and standard deviations of the dependent variables in the secretary problem.

	Standard SP						Punishment sensitive SP					
	Depressed (*N* = 20)			Healthy (*N* = 20)			Depressed (*N* = 21)			Healthy (*N* = 21)		
Measure	*M*	SD	Mdn	*M*	SD	Mdn	*M*	SD	Mdn	*M*	SD	Mdn
Search length	23.78	6.17	23.50	23.26	6.76	24.00	21.75	4.98	18.00	21.38	6.49	20.00
Relative rank	2.83	1.62	1.00	2.43	1.03	2.00	1.93	0.72	1.00	2.15	0.77	1.00
Performance	34.97	2.57	37.00	35.62	1.17	36.50	35.89	2.59	37.00	35.44	2.27	37.00
Goal	6.69	4.00	5.50	4.39	2.70	5.00	7.28	7.89	3.00	11.00^a^	9.79	8.00^b^

a One participant was excluded due to more than 10% missing values (*N* = 20).

b Three participants were excluded due to more than 10% missing values (*N* = 18).

Concerning the means, a 2 × 2 between-subjects ANOVA with the independent variables health status and condition showed no significant main effect or interaction for the performance measure or the search length, all *F*’s < 2.1. For the mean relative rank, a significant main effect of condition emerged, *F*(1, 78) = 6.02, *p* < 0.05, *η*^2^ = 0.07. The mean relative rank was higher for participants in the standard SP condition compared with the participants in the punishment sensitive condition. In the case of self-reported goals, the main effect of condition was also significant, *F*(1, 77) = 5.76, *p* < 0.05, *η*^2^ = 0.07. Participants in the standard SP condition declared the need to perform better to be satisfied with themselves than participants in the punishment sensitive condition. Furthermore, a Condition × Health Status interaction emerged, *F*(1, 77) = 4.00, *p* < 0.05, *η*^2^ = 0.05. In the punishment sensitive condition, the healthy participants declared that they were more satisfied with themselves after a worse performance than the participants with MDD. In the standard SP condition, interestingly, this pattern was the other way around. All other main effects and interactions were not significant, all *F*’s < 1.8. Nonparametric versions of the ANOVA (i.e., Kruskal-Wallis) yielded similar results; the only difference that emerged was that the main effect of condition on relative rank was not significant, *χ*^2^(3, *N* = 82) = 4.96, *p* = 0.18.

We repeated the analyses with the median of the performance measure and the median of the relative rank. Interestingly, a significant main effect of condition emerged for performance, *F*(1, 78) = 4.71, *p* < 0.05, *η*^2^ = 0.06. Participants in the punishment sensitive condition had higher performance scores than participants in the standard SP condition. The same main effect of condition emerged also for relative rank, *F*(1, 78) = 11.20, *p* < 0.01, *η*^2^ = 0.13. The median value of the relative rank was lower in the punishment sensitive compared with the standard SP condition. No other significant main effect or interaction emerged, all *F*’*s* < 2.5. Again, the results of the nonparametric approaches were consistent with these aforementioned results.

### Additional Analyses

As we did not find any significant differences between healthy and clinically depressed participants, we decided to conduct Bayes factor analyses. A Bayes Factor measures the amount of evidence for a hypothesis compared to another hypothesis by relating the posterior probabilities of the two hypotheses given the data (see Rouder et al., 2009, for an introduction). In the present case, it allowed us to measure the evidence for the null hypothesis (i.e., no differences between healthy and clinical depressed participants) in comparison to the alternative hypothesis (i.e., there are significant differences between the two samples). The results showed moderate evidence for the null hypothesis for all dependent measures, minimal BF_01_ = 3.72, maximal BF_01_ = 4.34.

A potential explanation for our results might be that we asked participants to complete 60 standard SPs rather than the 30 standard SPs von Helversen et al. (2011) used. To test this explanation, we conducted additional analyses by computing all models again but with the dependent variables computed across the first 30 trials (see Table 3). However, again, no significant differences between the healthy participants and participants with MDD occurred for any of the performance measures, all *F*’s < 1.50. Finally, we also looked for potential effects of antidepressant medication. However, independent *t*-tests showed that there were no significant differences between participants with MDD who used or did not use medication on any of the performance measures (see Table 4; all *t*’s < 1.39).

**Table 3:** Means, medians, and standard deviations of the dependent variables in the standard secretary problem split into two blocks of 30 trials.

	First block						Second block					
	Depressed (*N* = 20)			Healthy (*N* = 20)			Depressed (*N* = 20)			Healthy (*N* = 20)		
Measure	*M*	SD	Mdn	*M*	SD	Mdn	*M*	SD	Mdn	*M*	SD	Mdn
Search length	23.37	6.82	23.25	22.83	6.70	22.25	24.87	6.40	24.75	24.23	7.49	25.00
Relative rank	2.93	1.70	1.50	2.34	0.90	2.00	2.97	1.84	1.75	2.68	1.32	2.00
Performance	34.49	2.99	37.00	35.47	1.33	36.25	35.10	2.94	37.00	35.55	1.34	36.50
Goal	6.60	4.10	5.50	4.79	2.92	5.00	6.72	4.57	6.00	4.57	2.52	5.00

**Table 4:** Means, medians, and standard deviations of the dependent variables in the experimental groups according to the status of antidepressant medication in the secretary problem.

	Standard SP						Punishment sensitive SP					
	Antidepressant medication (*N* = 12)			No medication (*N* = 8)			Antidepressant medication (*N* = 9)			No medication (*N* = 12)		
Measure	*M*	SD	Mdn	*M*	SD	Mdn	*M*	SD	Mdn	*M*	SD	Mdn
Search length	24.04	7.14	24.00	23.39	4.79	23.00	21.50	6.72	19.00	21.94	3.47	18.00
Relative rank	2.98	1.93	1.00	2.62	1.08	1.50	2.04	0.92	1.00	1.84	0.56	1.00
Performance	34.96	2.95	37.00	35.00	2.06	37.00	35.00	3.83	37.00	36.56	0.63	37.00
Goal	6.33	2.94	7.00	7.22	5.42	5.00	6.89	7.46	2.00	7.58	8.51	3.50

### Correlations Between Performance Measures and Personality Variables

Fluid intelligence was positively related to mean search length and median search length, overall (*r* = 0.39, *p* < 0.01; *r* = 0.39, *p* < 0.01), for the healthy participants (*r* = 0.41, *p* < 0.01; *r* = 0.42, *p* < 0.01), and also for the participants with MDD (*r* = 0.37, *p* < 0.05; *r* = 0.37, *p* < 0.05). Furthermore, fluid intelligence was positively related to mean performance for all participants (*r* = 0.25, *p* < 0.05), but median performance was not (*r* = 0.11, *p* = 0.31). There also was no significant correlation between fluid intelligence and mean or median performance for participants with MDD (*r* = 0.27, *p* < 0.10; *r* = 0.12, *p* = 0.44) or healthy participants (*r* = 0.24, *p* = 0.13; *r* = 0.11, *p* = 0.51). Moreover, we found a positive correlation between neuroticism and the mean self-reported goals (*r* = 0.34, *p* < 0.05) for the healthy participants. No other effects were significant. The same results were obtained when we used Kendall’s tau instead of the product-moment correlation.

## Discussion

The goal of the present study was to replicate the results of von Helversen et al. (2011) showing that patients with MDD outperformed healthy participants in the sequential decision-making task SP. We also wanted to extend their findings by testing whether a hyposensitivity to reward or a hypersensitivity to punishment is responsible for the better performance of clinically depressed individuals. However, and in contrast to the results of von Helversen et al. (2011), we did not find any significant performance differences between participants with MDD and healthy control participants. Also, there was no correlation between depression and performance. Our hypothesis concerning the sensitivity to negative feedback could also not be confirmed.

With regard to the associations between the Big Five personality traits and performance in SP, we only found that for healthy participants neuroticism was correlated with self-reported goals. However, given the high number of correlations tested and given the mean differences in neuroticism between healthy and clinically depressed participants, we think that this finding has to be interpreted with caution. Thus, similar to earlier research (Brand and Altstötter-Gleich, 2008), we did not find strong evidence that personality is related to performance in sequential decision-making tasks.

Finally, fluid intelligence, which refers to reasoning and problem-solving, was associated with search length and performance, indicating that the higher the fluid intelligence of the participants the better the performance in the SP. Interestingly, this relationship occurred for all participants as well as for each of the two subsamples, indicating the central role of cognitive abilities in sequential decision-making.

In summary, depression had no effect on decision performance in our study as we did not find any significant performance differences between healthy participants and participants with MDD. Furthermore, while personality traits were unrelated to decision performance, fluid intelligence was positively associated with it.

### Explanations for the Inconsistent Results

A number of explanations for the nonreplication of von Helversen et al.’s findings are possible. An important difference between von Helversen et al.’s (2011) participants and ours was that our participants were not hospital patients but ambulatory patients. Thus, even though the BDI-II scores in our study were as high as they were in von Helversen et al.’s (2011) study, the quality of depression in our sample may have been less extreme.

Another explanation might be the influence of intelligence. As stated by Burns et al. (2006), general intelligence influences performance in the SP. In our study, we were able to show that participants with higher fluid intelligence waited longer to choose an applicant. Thus, intelligence might be more important for the strategy that was chosen and therefore for performance than the severity of depression. In von Helversen et al.’s (2011) study, intelligence was not controlled for, so there is a possibility that the sample with MDD in their study might have had higher intelligence scores than the healthy sample.

Another explanation concerns our healthy control participants. In von Helversen et al.’s (2011) study, the control sample performed worse than the sample of individuals with MDD. More importantly, the mean performance of von Helversen et al.’s control sample was worse than the average performance of our control participants. Hence, the control participants in our study might have been more motivated (e.g., they volunteered because of personal interest), resulting in no performance differences from the sample with MDD. Finally, methodological reasons such as the small sample size or the low reliability of the personality measures may have contributed to the inconsistent results and null effects found in our study with regard to the relationship between personality traits and SP performance.

### Implications and Questions for Future Research

The effect of depression on decision-making is a controversial issue. Consistent with the diagnostic criteria for depression in the DSM-5 (American Psychiatric Association, 2013), many studies have shown that depression regularly leads to diminished decision-making performance in comparison with healthy persons (e.g., Nestler and Carlezon, 2006; Cella et al., 2010). However, other studies have also reported a better performance of participants with MDD compared with healthy participants (e.g., Smoski et al., 2008), and even other studies showed that there are no performance differences between the two samples (e.g., Dalgleish et al., 2004; see also Kyte et al., 2005, for similar results using the IGT). The results of our study are consistent with these latter studies by showing no reliable performance differences between clinically depressed and healthy individuals.

We hypothesized that when sensitivity to punishment is enhanced in individuals with MDD, this may additionally increase their performance (e.g., Smoski et al., 2008). Contrary to our hypothesis, punishment sensitivity had an overall effect on performance regardless of the actual health status of the participants. This result seems to be inconsistent with the findings of Campbell and Lee (2006) who could not find any effect of feedback on the performance in the SP. However, they did not use specifically negative feedback (but rather, for example, a simple feedback if the decision made was “correct” or “wrong”). On a more general level, the main effect of punishment sensitivity is consistent with the hypothesis that individual’s decisions will tend to change when they are given negative feedback (e.g., Wofford and Goodwin, 1990) and that negative feedback results in a bad mood, which in turn may promote a more accommodative, attentive, and externally focused thinking strategy (Forgas, 2017). Nevertheless, we believe that it is an interesting task for future research to further investigate the effect of feedback (be it negative, positive or rather neutral) on the performance in a sequential decision-making task such as the SP. In this course, it might also be interesting to further investigate the way decisions are made (e.g., if the somatic marker hypothesis plays a potential role, if heuristics are rather used, etc.). Similarly, we think that it would be interesting to replicate these findings with other decision-making tasks such as the Pandora’s search problem^8^ (Weitzman, 1979) as another sequential decision-making task or the “Water Purification Plant”^9^ (WPP; Gonzalez, 2005) task as another sort of dynamic decision-making task to see if our results could be replicated with these tasks or if it is a specific effect for the SP.

Another interesting task for future research might be to investigate the role of rumination. Whitmer et al. (2012), for example, found that depressive rumination leads to reduced sensitivity to punishment and thus to diminished performance (Steffens et al., 2001; Must et al., 2006). We note that the severity of rumination might also explain why we could not replicate von Helversen et al.’s (2011) results as there might be difference in rumination between our clinically depressed sample and the sample of these authors. This might also explain why we did not find an effect of the punishment sensitivity manipulation. Therefore, we believe that future experiments should assess patients’ amount of rumination and should relate it to their performance.

It might also be interesting for future research to explore other clinical groups. In particular, patients who have been diagnosed with a generalized anxiety disorder (GAD) should be analyzed. Mueller et al. (2010) administered the IGT to patients suffering from GAD. According to the American Psychiatric Association (2013), this disorder is characterized by “excessive anxiety and worry” and may include, for example, difficulties in concentrating. The authors found that persons with a high score on worrying learned to avoid decisions with a high probability of long-term loss faster than controls did. According to the authors, this means that “GAD is characterized by enhanced processing of potential future losses rather than sensitivity to large short-term loss” (Mueller et al., 2010). We believe that this hypothesis should also be examined for the SP.

Finally, individuals with depression typically complain about diminished decision-making. Given the present findings, it might be the case that this is more a subjective feeling that is related to low self-esteem (American Psychiatric Association, 2013). This is important insofar as it is this feeling that may result in indecisiveness as well as insecurity and thus in diminished performance in some sort of decision-making tasks. For instance, when the clinically depressed individuals are forced to make decisions in a rather simple situation, they are, at least respective to sequential decision-making, able to make decisions that are equally good as the decisions of healthy individuals. We believe that this self-esteem hypothesis should be investigated in future research.

## Conclusion

The present study could not replicate the results of von Helversen et al. (2011). We were not able to detect any significant differences between the performances of healthy and participants with MDD in a sequential decision-making task (i.e., the SP) or in a modified version of the SP that involved feedback (punishment sensitive). Furthermore, although fluid intelligence was related to performance in the sequential decision-making task, the Big Five traits were not. Overall, our results should be taken as evidence that the relationship between depression and sequential decision-making is complex, highlighting that more research is needed in this important domain.

## Ethics Statement

All procedures performed in studies involving human participants were in accordance with the ethical standards of the institutional research committee and with the 1964 Helsinki declaration and its later amendments or comparable ethical standards. Informed consent was obtained from all individual participants included in the study.

## Author Contributions

MS and SN designed the study. MS undertook the statistical analysis, searched for literature, and wrote the first draft of the manuscript. SN and BE corrected the first draft and completed the manuscript. All authors contributed to and have approved the final manuscript.

### Conflict of Interest Statement

The authors declare that the research was conducted in the absence of any commercial or financial relationships that could be construed as a potential conflict of interest.

## Footnotes

^1^In DDM tasks an important problem is often the impact of delayed feedback. In order to make good decisions or to improve them, it is necessary to know which result is due to which part of the own decision-making process. In DDM tasks (as in real life situations) this feedback often occurs delayed. For a thorough discussion of this topic, see Brehmer (1989).

^2^The TSP task in this paradigm is to define a certain sequence for visiting different locations without visiting the same two times, except the location you have started from. The distance should be as small as possible and the first location should also be the last one.

^3^https://osf.io/4cwqn/?view_only=499496bbc4714c9d8766e0dd6d14b4a3

^4^100 Cents = 1 Euro.

^5^We note that in case of the BFI-S subscales, test-retest correlations are more adequate measures of their reliabilities than internal consistency coefficients because the three items tap very different aspects of the very broad Big Five dimensions. In line with this reasoning, the test-retest stabilities of brief Big Five measures are usually satisfying (e.g., Gosling et al., 2003).

^6^Crystallized intelligence and fluid intelligence are components of general intelligence with crystallized intelligence referring to a person’s culture specific knowledge and verbal abilities, and fluid intelligence referring to the person’s reasoning and problem-solving abilities (see Cattell, 1971).

^7^Before analyzing our results, we had to exclude a total amount of 105 trials (in addition to the 258 excluded trials in which the 40th applicant was chosen) out of the 4,920 due to technical problems during the task. Interestingly, clinically depressed and healthy participants did not differ in the amount of trials in which they stopped at the 40th applicant [*t*(80) = −1.09, *p* = 0.28].

^8^In this task, the decision maker is confronted with a certain number of closed boxes. Each box contains a potential individual reward that is independent of the rewards in the other boxes. To open a box and thus to find out about the specific reward, has a certain cost on the other hand. Also, the reward of a box is disclosed not till a short, varying delay to the decision maker. He always has to decide whether or not he wants to open the next box. If he chooses to stop searching, he gets the reward so far uncovered. If he wants to continue searching, he has to select the next box that should be opened and he has to pay the fee for opening it. Contrary to the SP, the sum of search costs is paid during search, whereas the reward is not known immediately but conveyed to the decision maker after search has been terminated.

^9^The WPP simulates a water distribution system with 23 tanks and is a computer-based, interactive, simulation of a real-world scheduling task (mail sorting in the United States Postal Service). The goal is to distribute all the available water in the system before various deadlines may expire. The WPP is categorized as a typical DDM task, because it involves all of the integral characteristics. It is a dynamic resource allocation task and the environment changes according to the actions of the decision maker and on its own. It is also a very complex task and also involves opaque variables.
